# Number-induced shifts in spatial attention: a replication study

**DOI:** 10.3389/fpsyg.2014.00987

**Published:** 2014-09-10

**Authors:** Kiki Zanolie, Diane Pecher

**Affiliations:** ^1^Department of Psychology, Erasmus University RotterdamRotterdam, Netherlands; ^2^Institute of Psychology, Leiden UniversityLeiden, Netherlands

**Keywords:** mental number line, attention, abstract concepts, concept representation, grounded cognition, SNARC effect, image schema

## Abstract

In a spatial attention paradigm, Fischer et al. (2003) showed that merely perceiving a number shifted attention according to the magnitude of the number. Low numbers shifted attention to the left and high numbers shifted attention to the right. This suggests that numbers are represented by the mental number line – a spatial image schema that is ordered from left to right with increasing magnitude. In six experiments, we used the spatial attention paradigm of Fischer et al. (2003) to investigate if and when such mental representations are activated. Participants detected visual targets that were preceded by low and high numbers. Between experiments we manipulated how participants processed the number. Participants either merely perceived the number, as in the experiments by Fischer et al. (2003) processed the number’s parity, or processed the number’s magnitude. Our results provide little support for the idea that numbers shift spatial attention. Only in one of the two experiments in which participants processed number magnitude did participants respond faster to targets in congruent locations (left for low magnitudes and right for high magnitudes) than in incongruent locations. In the other five experiments number magnitude did not affect spatial attention. This shows, in contrast to Fischer et al.’s (2003) results, that the mental number line is not activated automatically but at best only when it is contextually relevant. Furthermore, these results suggest that image schemas in general may be context-dependent rather than fundamental to mental concepts.

## NUMBER-INDUCED SHIFTS IN SPATIAL ATTENTION: THE NECESSITY OF MAGNITUDE INFORMATION

Dehaene et al. (1990, 1993) demonstrated that low numbers are associated with faster left side responses and high numbers are associated with faster right side responses, an effect also known as the spatial–numerical association of response codes (SNARCs). These findings suggest that whenever a number is perceived an internal spatial representation of magnitude is automatically activated, in the form of a horizontally oriented mental number line, with increasing magnitude from left to right. The SNARC effect is thought to arise through activation of spatial codes associated with the magnitude of the number.

But what exactly is represented by the mental number line? The mental number line orders numbers according to magnitude by spatially placing them on a horizontal line. Thus, the mental number line is an image schema of number *magnitude*. However, magnitude does not represent number meaning completely. People also have other knowledge about numbers, such as what constitutes a round number or a number’s parity. Such knowledge is not captured by the mental number line. Therefore the question is whether the mental number line is activated automatically whenever people represent numbers or only when magnitude information is relevant. Some results from SNARC studies suggest that the effect depends on task requirements. When subjects were asked to perform an orientation discrimination task on a line or triangle superimposed on a digit a SNARC effect was found (Fias et al., 2001; Lammertyn et al., 2002). However, this effect was not consistent over tasks and disappeared when participants were asked to report the color of the digit, or to identify the shape superimposed on the digit.

Moreover, several researchers argue that the SNARC effect is at least partly due to response-related activation of spatial information (Otten et al., 1996; Fischer, 2003; Keus and Schwartz, 2005; Keus et al., 2005; Gevers et al., 2006; Daar and Pratt, 2008). Participants usually make left/right responses to the numbers, which creates a direct and task-relevant link between number and spatial response codes. To avoid response effects, a better way to test activation of mental image schemas is to look at modulation of performance on an irrelevant target (e.g., Fischer et al., 2003; Richardson et al., 2003; Meier and Robinson, 2004; Pecher and Boot, 2011; Zanolie et al., 2012). Fischer et al. (2003) used a simple target detection task in which the target locations were on the left or right side of the fixation point. Prior to presentation of the target participants perceived a number that was low or high in magnitude (1, 2 or 8, 9) in the center of the screen. The number did not predict the target location and thus was irrelevant for the task. Importantly, because participants responded only with their preferred hand by pressing the spacebar, there was no interference of spatial response codes. In addition, the target stimulus was a white circle, whereas in classical SNARC tasks some attribute of the number (e.g., parity) is the relevant stimulus. Fischer et al. (2003) showed that participants were faster to detect a target on the left side of the visual field after perceiving a low number than a high number, and faster to detect a target on the right side of the visual field after perceiving a high number than a low number. Based on these results Fischer et al. (2003) claimed that mere observation of numbers automatically activates spatial representations associated with number meaning, which in turn influences the allocation of attention in the visual field. These results suggest that whenever people perceive numbers they activate a spatial, horizontal mental number line.

The results of Fischer have widespread implications for the question how we represent concepts. The idea of a mental number line may be a good example of a metaphorical mapping that grounds abstract concepts in concrete, spatial domains, as proposed in the conceptual metaphor theory (Lakoff and Johnson, 1980, 1999; Lakoff, 1987; Gibbs, 1994). In this theory, mental concepts take their structure from concrete image schemas, which are dynamic patterns of multi-modal activation that emerge from recurring perceptual and action experiences (Johnson, 1987; Hampe and Grady, 2005) such as *vertical orientation* or *balance*. According to the conceptual metaphor theory, image schemas are fundamental for the representation of abstract concepts.

A metaphor explanation for *number* representation is supported by studies showing a strong association between number magnitude and space. As such, the results of Fischer et al. (2003) suggest that an image schema in the form of a mental number line is activated when people perceive a number. On this account, the mental number line might be essential for the representation of numbers. However, to our knowledge, there have been only two published reports of exact replications of the Fischer et al. (2003) study in which *all* aspects of the experimental design were kept precisely the same and reported the same results (Ristic et al., 2006; Dodd et al., 2008). Dodd et al. (2008) did not find attention effects with other ordinal sequences such as letters, days and months, showing that the attention effect was specific for numbers. Only when participants made an ordinal relevant decision on the cue (letter, day, or moth) after target detection an attention effect was found. Furthermore, in a modified version of the Fischer et al. (2003) experiment Galfano et al. (2006) replicated the number-attention effect. Other replication attempts of Fischer et al.’s (2003) in which one or more aspects of the experimental design were changed, have not always been successful (Galfano et al., 2006; Ristic et al., 2006; Casarotti et al., 2007; Bonato et al., 2008). For example, shorter presentation time of the number cues, number cue trials intermixed with arrow cue trials, and lower ratios of valid vs. invalid trials (Bonato et al., 2008) resulted in null effects. Casarotti et al. (2007) and Bonato et al. (2008) performed conceptual replications of Fischer et al. (2003) but found no number-induced attention effect, although Casarotti et al. (2007) did show that naming the numbers leads to a number-induced attention effect. In contrast, other studies obtained effects on attention-related ERP components but failed to show behavioral effects (Salillas et al., 2008; Ranzini et al., 2009). Such mixed findings suggest that merely perceiving numbers may not activate the mental number line in every context. Additional evidence shows that instructions can cause the opposite spatial effects (Galfano et al., 2006; Ristic et al., 2006), which suggests that the left-to-right mental number line is easily overruled by alternative spatial image schemas.

A possible explanation for these mixed findings is that people do not always activate magnitude information when they process numbers, at least not to the same extent. The activation of magnitude likely depends on its relevance in the context, with stronger activation if magnitude is relevant and weaker or no activation if magnitude is not relevant. Research on the flexibility of concepts has shown that representations contain more context-relevant than context-irrelevant features (Barclay et al., 1974; Anderson et al., 1976; Barsalou, 1982; Tabossi, 1988; Zeelenberg et al., 2003; Pecher et al., 2004, 2007). Given the evidence that concepts are context-dependent, it seems reasonable to assume that magnitude information is also context-dependent and thus might not be fully activated whenever a number is perceived. As a consequence, activation of the mental number line image schema should also be context-dependent.

In the current study we attempted to replicate Fischer et al.’s (2003) findings twice. Their paper has constituted an important test of the idea that the mental number line is activated automatically and is still considered an important paper. For example, the paper has been cited 256 times in the 11 years since it was published, and received its highest number of citations in the last years (33 and 31 citations in 2012 and 2013 in ISI web of knowledge, respectively). However, as reviewed above, there have been failures to replicate their finding albeit with different experimental procedures than in the original study. Therefore, an exact replication is in order. In addition, to test if context-dependency might explain previous mixed findings we investigated the effect of context on activation of the mental number line image schema by directly manipulating the importance of magnitude information. We conducted a series of experiments in which we varied task-related processing of numbers at three levels: not necessary, necessary but magnitude-irrelevant, and necessary and magnitude-relevant. The question was to which degree numbers have to be processed in order to activate the mental number line image schema and induce a number-induced visual spatial attention effect. In Experiment 1 we tried to replicate the findings of Fischer et al. (2003) by administering the exact same paradigm in which participants perceived a number that was irrelevant for the target detection task. In Experiment 2 we additionally asked participants to report the parity of the number after each trial. We reasoned that reporting the parity of the number required participants to actively process the number but would keep magnitude information irrelevant. If activation of magnitude information is context-dependent, we would expect little activation of the mental number line. However, in classical SNARC studies parity judgments often induce spatial response effects, suggesting that magnitude information might be context-independent. In Experiment 3 we asked the participant to report the magnitude of the number by judging whether the number was higher or lower than 5. We expected to find a number-induced attention effect in this experiment since the participant had to explicitly process magnitude, thereby activating the mental number line image schema.

In order to ensure enough power we doubled the number of participants as tested by Fischer et al. (2003). All other methods and analyses were identical (if possible) to the experiment conducted by Fischer et al. (2003). Below, we report these three experiments. To anticipate the results, we did not replicate Fischer et al.’s (2003) effect of number magnitude on spatial attention, unless number magnitude was task-relevant (Experiment 3). Of course, one failure to replicate does not invalidate the original result. However, when several attempts to replicate the original finding fail to do so, this informs us that the original effect may at least be fragile. Therefore, we replicated our set of three experiments exactly as reported below, leading to a second exact replication of Fischer et al.’s (2003) experiment.

## EXPERIMENT 1

### METHOD

#### Participants

Twenty students of the Erasmus University Rotterdam participated for course credit.

#### Stimuli

The number set consisted of the Arabic digits 1, 2, 8, and 9. The target stimulus was a small circle, which was presented at a 7° visual angle from fixation in one of two placeholders (rectangular frames slightly larger than the circle) to the left or right of a central fixation point. All stimuli were white on a black background. Participants were seated ∼70 cm from the screen.

#### Procedure

**FIGURE 1:**
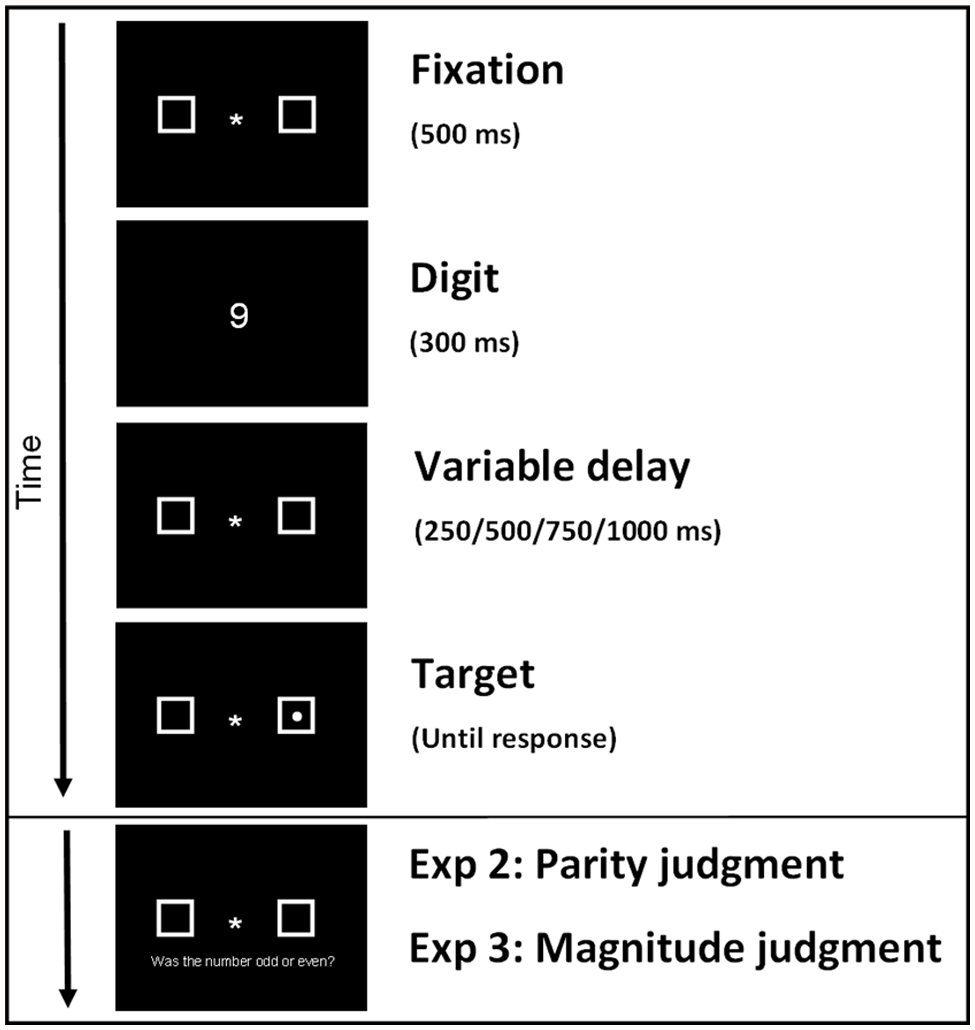
**The trial sequence started with a 500 ms fixation cross, followed by a 300 ms digit (1, 2, 8, 9) display, and a variable delay of 250, 500, 750, or 1000 ms.** Then a target was presented randomly in one of the two placeholders on 80% of all trials. Participants had to respond as fast as possible by pressing the spacebar when they detected the target. In Experiment 2 and 3 participants decided after target detection whether the previously seen digit was odd or even (Experiment 2 and Replication Experiment 2) or whether the digit was higher or lower than 5 (Experiment 3 and Replication Experiment 3).

The paradigm followed that of Fischer et al. (2003), see **Figure 1** for an overview of a trial sequence. A fixation sign was presented for 500 ms, followed by one of four digits (1, 2, 8, or 9) for 300 ms. The digit was replaced by the fixation with a random duration of 250, 500, 750, or 1000 ms (as in Fischer et al., 2003), after which the target was presented randomly in one of the two placeholders on 80% of all trials. Participants had to respond as fast as possible by pressing the spacebar with their preferred hand when they detected the target. Participants were instructed to maintain fixation at the center of the screen throughout the experiment, either at the fixation sign or at the digit. They were informed that the digits (1, 2, 8, or 9) did not predict the target location and were irrelevant for the detection task. Participants first received a practice session of 20 trials. The experiment consisted of six blocks separated by self-paced breaks. Each block consisted of 160 trials, in which 128 target trials and 32 catch trials (no-target) were randomly presented, resulting in 960 trials total.

### RESULTS AND DISCUSSION

Trials with incorrect responses to the target were excluded from the analysis (1.04%). Trials with reaction times more than 2.5 standard deviations faster or slower than the subject’s mean reaction time were also excluded from analysis (2.92%).

**FIGURE 2:**
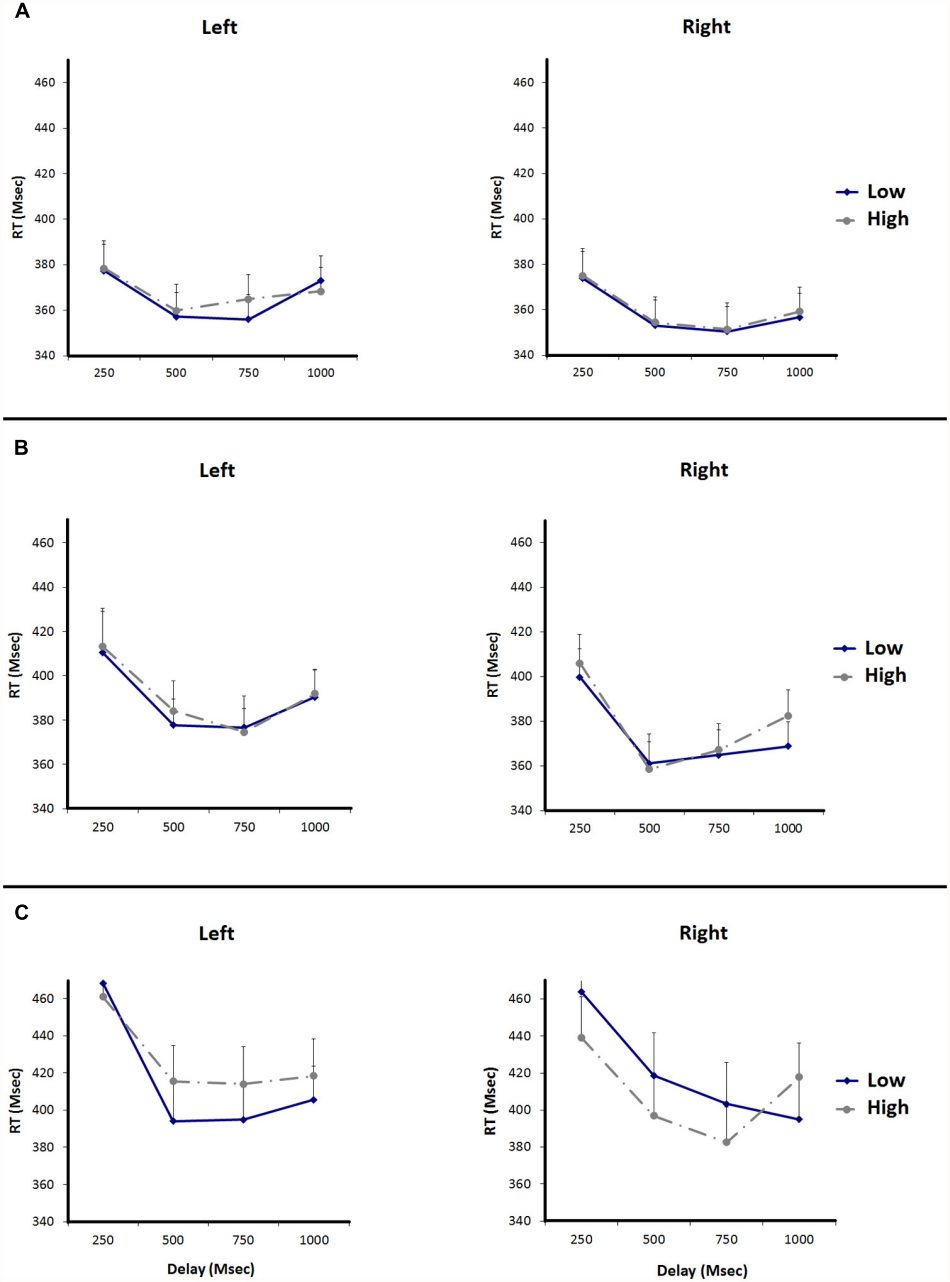
**Reaction times for the target detection task of experiments 1, 2, and 3.** Error bars represent standard errors of the mean difference between adjacent data points. **(A)** Shows the RTs of Experiment 1, **(B)** shows the RTs of Experiment 2, and **(C)** shows the RTs of Experiment 3.

In **Figure 2A**, the reaction times to the target are plotted. In the left panel reaction times to targets presented at the left are plotted, in the right panel reaction times to targets presented at the right are plotted. The mean reaction times on the target task were submitted to a 2 (Magnitude: high vs. low digit) × 2 (Side: left vs. right) × 4 (Delay: 250, 500, 750, or 1000 ms) repeated measures analysis of variance (ANOVA). We found main effects for Delay, *F*(3,57) = 15.10, *p* < 0.0001 and Side, *F*(1,19) = 5.67, *p* = 0.028. Participants responded faster to targets after delays of 500 and 750 ms, compared to delays of 250 and 1000 ms. Participants also responded faster to targets on the right compared to targets on the left. The main effect for Magnitude was not significant, *F* < 1. The Magnitude × Side interaction effect did not reach significance, *F*(1,19) = 0.03, *p* = 0.863. Because the ANOVA *p*-values cannot be used to provide evidence in favor of the null hypothesis (it can only be used to reject it), we further analyzed the interaction effects between magnitude (high vs. low number) and side (left vs. right) using the Bayesian information criterion (BIC; see Wagenmakers, 2007; Masson, 2011). The posterior probability favoring the null hypothesis was *p*_BIC_(H_0_ | D) = 0.81 for the two-way interaction between magnitude and side. BIC values between 0.75 and 0.95 should be considered positive evidence for a hypothesis (Wagenmakers, 2007; Masson, 2011). Thus, it appears that merely perceiving numbers did not affect spatial attention. This could be because magnitude information was not activated, or magnitude information was not relevant for the task, or the number was not processed at all. None of the other interaction effects were significant (all *p*s > 0.05).

## EXPERIMENT 2

From the results of Experiment 1 it is clear that merely perceiving numbers did not result in a number-induced attention effect in a mental number line congruent fashion. It is possible that participants completely ignored the numbers since they were task-irrelevant. In Experiment 2 numbers were made relevant by asking participants to make a parity judgment. In the original SNARC effect, making a parity judgment leads to faster RTs for the left hand when a low number is presented and faster RTs for the right hand when a high number is presented. At the same time, however, parity is unrelated to magnitude and thus to a number’s position on the mental number line. However, if magnitude is activated automatically and independent of context, an interaction should be found between number magnitude and target position.

### METHOD

#### Participants

Twenty-seven students of the Erasmus University Rotterdam, who did not participate in Experiment 1, participated for course credit. Two participants were excluded due to a high percentage of errors in the catch trials (>8%) and one participant was excluded due to high percentage of errors made in the parity judgment task (12.71%), resulting in 24 remaining participants.

#### Stimuli and procedure

All stimuli were the same as in Experiment 1. The same procedure as in Experiment 1 was conducted, with the addition of an interval of 200 ms after each target response followed by a display instructing participants to make a parity judgment on the digit. Participants responded to the target by pressing the spacebar with their right hand. For making the parity judgment participants responded with their left hand with their middle- and index finger by pressing the “*z”* and “*x”* keys for odd or even. The assignment of response keys to parity was counterbalanced across participants. In order to keep the procedure as similar as possible to that of Experiment 1 responses to the target were always made by the preferred hand, which in all cases was the right hand (see also Fischer et al., 2003). Participants first received a practice session of 20 trials. The experiment consisted of three blocks separated by self paced breaks. Each block consisted of 160 trials, in which 128 target trials and 32 catch trials (no-target) were presented in random order, resulting in 480 trials total.

### RESULTS AND DISCUSSION

Trials with incorrect responses to either the target (0.90%) or parity judgment (4.04%) were excluded from the analysis. Trials with reaction times more than 2.5 standard deviations faster or slower than the subject’s mean reaction time were also excluded from analysis (3.44%).

In **Figure 2B**, the reaction times to the target are plotted. In the left panel reaction times to targets presented at the left are plotted, in the right panel reaction times to targets presented at the right are plotted. The mean reaction times on the target task were submitted to a 2 (Magnitude: high vs. low digit) × 2 (Side: left vs. right) × 4 (Delay: 250, 500, 750, or 1000 ms) repeated measures ANOVA. We found main effects for Delay, *F*(3,69) = 27.40, *p* < 0.0001, and Side, *F*(1,23) = 18.35, *p* < 0.0001. Participants responded faster to targets after delays of 500–750 ms, compared to delays of 250–1000 ms. Participants responded faster to targets on the right side compared to targets on the left. The main effect for Magnitude was not significant, *F*(1,23) = 0.78, *p* = 0.388. Again, we did not find a Magnitude × Side interaction effect, *F*(1,23) = 0.17, *p* = 0.686. Number magnitude did not affect visual spatial attention. None of the other interactions were significant (all *p*s > 0.05). We further analyzed the interaction effects between magnitude and side using the BIC. The posterior probability favoring the null hypothesis was *p*_BIC_(H_0_ | D) = 0.82 for the two-way interaction between magnitude and side. This shows that there is more evidence for the null hypothesis than for the alternative hypothesis.

In order to test whether participants showed a SNARC effect on the parity decision we performed a 2 (Magnitude: high vs. low digit) × 2 (Side: left vs. right) × 4 (Delay: 250, 500, 750, or 1000 ms) repeated measures ANOVA with Group (congruent vs. incongruent response mapping) as between subjects factor (see Priftus et al., 2006). We did not find a significant Magnitude × Side × Group interaction, *F*(1,22) = 0.01, *p* = 0.975. Also, the Magnitude × Side interaction remained non-significant, *F*(1,22) = 0.16, *p* = 0.696.

## EXPERIMENT 3

From Experiment 2 it is clear that processing the number on parity is not enough to obtain number-induced attention effects. This is consistent with the idea that magnitude representation is context-dependent. In Experiment 3 participants were required to make a magnitude judgment in order to make magnitude task-relevant. If magnitude information is represented by a spatial image schema we would expect a number-induced attention effect.

### METHOD

#### Participants

Twenty-two students of the Erasmus University Rotterdam, who did not participate in Experiment 1 or 2, participated for course credit. Two participants were excluded due to a high percentage of errors in the catch trials (>10%), resulting in a total of 20 participants.

#### Stimuli and procedure

All aspects of the experiment were exactly the same as in Experiment 2, with one exception. Instead of a parity judgment, participants were asked whether the digit was higher or lower than 5.

### RESULTS AND DISCUSSION

Trials with incorrect responses to either the target (1.13%) or magnitude judgment (3.25%) were excluded from the analysis. Trials with reaction times more than 2.5 standard deviations faster or slower than the subject’s mean reaction time were also excluded from analysis (5.62%). In **Figure 2C**, the reaction times to the target are plotted. In the left panel reaction times to targets presented at the left are plotted, in the right panel reaction times to targets presented at the right are plotted. The reaction times on the target task were submitted to a 2 (Magnitude: high vs. low digit) × 2 (Side: left vs. right) × 4 (Delay: 250, 500, 750, or 1000 ms) repeated measures ANOVA. As expected, we found an interaction effect for Magnitude × Side, *F*(1,19) = 5.65, *p* = 0.028. After seeing a low number participants were faster to respond to a target on the left side compared to a target on the right side, and after seeing a high number participants were faster to respond to a target on the right side compared to a target on the left side. We found a main effect for Delay, *F*(3,57) = 20.29, *p* < 0.0001. Delay did not interact with magnitude or side. The main effects for Magnitude and Side were not significant (*Fs* < 1). None of the other interactions were significant (all *p*s > 0.05).

To test at which delays the Magnitude × Side interaction effect was significant we first performed a 2 (Magnitude: high vs. low) × 2 (Side: left vs. right) repeated measures ANOVA for each delay. We only found a significant Magnitude × Side interaction effect at delays 500 and 750 ms, respectively *F*(1,19) = 6.12, *p* = 0.023 and *F*(1,19) = 5.24, *p* = 0.034. For delays 250 and 1000 ms we found *F*s < 1. Then we performed *post hoc* one-tailed *t*-tests at delays 500 and 750 ms. We found a significant difference for targets presented at the left at delay 750 ms, *t*(1,19) = 2.00, *p* = 0.06. However, this would not be significant when tested two-tailed. At a delay of 500 ms we did not find a significant difference for targets presented at the right *t*(1,19) = 1.59, *p* = 0.12. All other *p*s > 0.162.

We further analyzed the interaction effects between magnitude and side using the BIC. The posterior probability favoring the alternative hypothesis was *p*_BIC_(H_1_| D) = 0.75 for the two-way interaction between magnitude and side, which provides positive evidence for a number-induced effect on spatial attention.

In order to test whether participants showed a SNARC effect on the magnitude decision we performed a 2 (Magnitude: high vs. low digit) × 2 (Side: left vs. right) × 4 (Delay: 250, 500, 750, or 1000 ms) repeated measures ANOVA with Group (congruent vs. incongruent response mapping) as between subjects factor (see Priftus et al., 2006). Interestingly, we found a significant Magnitude × Side × Group interaction, *F*(1,18) = 6.03, *p* = 0.024. Participants were faster to make the magnitude decision when the response mapping was congruent (right finger – high number and left finger – low number) compared to incongruent (right finger – low number and left finger – high number). Although this comparison, by necessity, is a between subjects comparison, Dehaene et al. (1993) in their original SNARC report also conducted a between subjects comparison to demonstrate the presence of number-space mappings. Also the Magnitude × Side interaction remained significant, *F*(1,18) = 7.27, *p* = 0.009. However, we should be cautious to draw conclusions from these data because the effects on the secondary task might be affected by the target detection task.

Additionally, we performed an overall analysis on the data from all three experiments; a 3 (Experiment: 1, 2, and 3) × 2 (Magnitude: high vs. low digit) × 2 (Side: left vs. right) × 4 (Delay: 250, 500, 750, or 1000 ms) repeated measures ANOVA. Of most interest is that we did find an Experiment × Magnitude × Side interaction effect, *F*(2,61) = 3.65, *p* = 0.032. This finding, combined with the BIC values of the three separate experiments, confirms that the effect of number magnitude on spatial attention was present in Experiment 3 but absent in Experiments 1 and 2. However, the Experiment × Magnitude × Side × Delay interaction did not reach significance, *F*(6,183) = 0.56, *p* = 0.76.

## REPLICATION OF EXPERIMENTS 1–3

In the three experiments described above, we tried to replicate the finding that merely perceiving a number affects spatial attention. We found that spatial attention was not affected by number magnitude. Even when participants processed the number in a parity judgment task, there was no effect on spatial attention. Only when participants processed number magnitude did we find that attention was directed to the left by low numbers and to the right by high numbers. Below we report the results of exact replications of our three experiments, including the exact replication of Fischer et al. (2003). We tested 24 participants in each experiment.

## REPLICATION EXPERIMENT 1

### RESULTS AND DISCUSSION

Trials with incorrect responses to the target were excluded from the analysis (5.4%). Trials with reaction times more than 2.5 standard deviations faster or slower than the subject’s mean reaction time were also excluded from analysis (2.94%).

**FIGURE 3:**
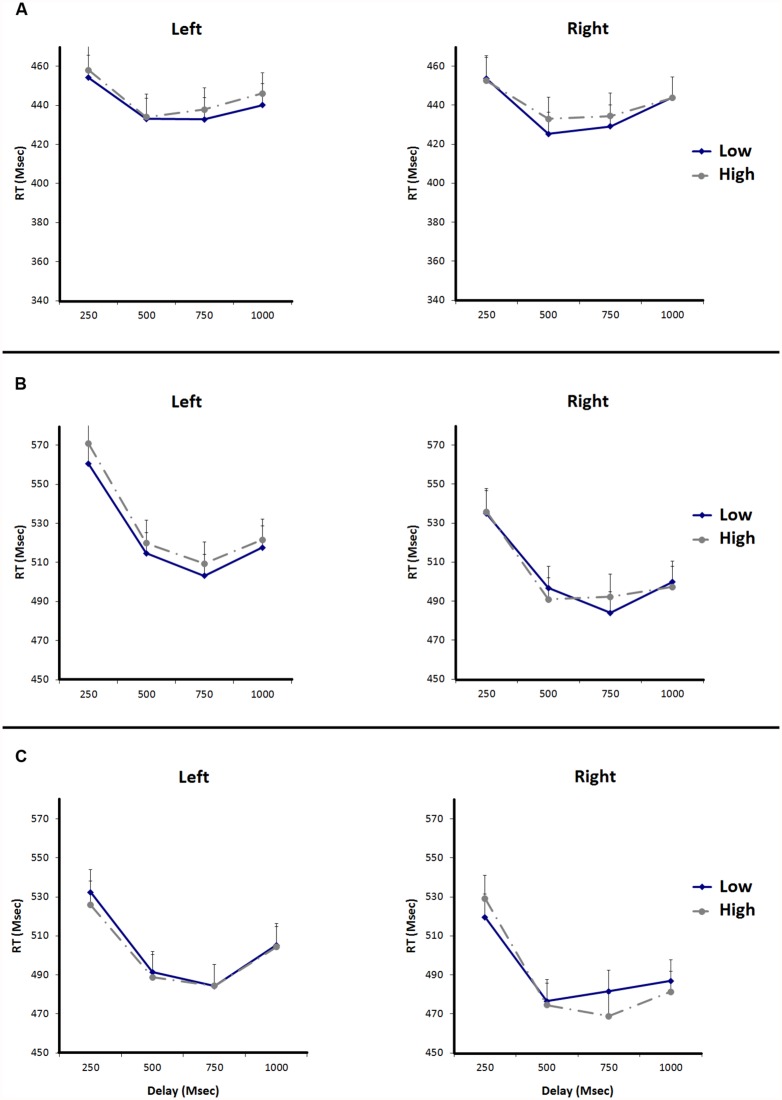
**Reaction times for the target detection task of replications experiments 1, 2, and 3.** Error bars represent standard errors of the mean difference between adjacent data points. **(A)** Shows the RTs of Replication Experiment 1, **(B)** shows the RTs of Replication Experiment 2, and **(C)** shows the RTs of Replication Experiment 3.

In **Figure 3A**, the reaction times to the target are plotted. In the left panel reaction times to targets presented at the left are plotted, in the right panel reaction times to targets presented at the right are plotted. The mean reaction times on the target task were submitted to a 2 (Magnitude: high vs. low digit) × 2 (Side: left vs. right) × 4 (Delay: 250, 500, 750, or 1000 ms) repeated measures ANOVA. We found main effects for Delay, *F*(3,69) = 37.68, *p* < 0.0001 and Magnitude, *F*(1,23) = 8.37, *p* = 0.008. Participants responded faster to targets after delays of 500 and 750 ms, compared to delays of 250 and 1000 ms. Participants also responded faster to targets after seeing low numbers compared to high numbers. The main effect for Side was not significant, *F* < 1, nor was the Magnitude × Side interaction effect, *F*(1,23) = 0.13, *p* = 0.722. The posterior probability favoring the null hypothesis was *p*_BIC_(H_0_ | D) = 0.82 for the two-way interaction between magnitude and side, providing positive evidence for the null hypothesis (Wagenmakers, 2007; Masson, 2011). None of the other interaction effects were significant (all *p*s > 0.05).

Also, when we performed an analysis of Experiment 1 and Experiment 4 combined using the BIC. The posterior probability favoring the null hypothesis was *p*_BIC_(H_0_ | D) = 0.86 for the two-way interaction between magnitude and side, confirming that there is more evidence for the null hypothesis than for the alternative hypothesis. With these results we replicated the results of Experiment 1, and confirm that merely perceiving numbers did not affect spatial attention.

## REPLICATION EXPERIMENT 2

### RESULTS AND DISCUSSION

Trials with incorrect responses to either the target (2.13%) or parity judgment (3.28%) were excluded from the analysis. Trials with reaction times more than 2.5 standard deviations faster or slower than the subject’s mean reaction time were also excluded from analysis (5.35%).

In **Figure 3B**, the reaction times to the target are plotted. In the left panel reaction times to targets presented at the left are plotted, in the right panel reaction times to targets presented at the right are plotted. The mean reaction times on the target task were submitted to a 2 (Magnitude: high vs. low digit) × 2 (Side: left vs. right) × 4 (Delay: 250, 500, 750, or 1000 ms) repeated measures ANOVA. We found main effects for Delay, *F*(3,69) = 48.91, *p* < 0.0001, and Side, *F*(1,23) = 19.78, *p* < 0.0001. Participants responded faster to targets after delays of 500, 750, and 1000 ms, compared to delays of 250 ms. Participants responded faster to targets on the right side compared to targets on the left. The main effect for Magnitude was not significant, *F*(1,23) = 1.17, *p* = 0.291. Again, we did not find a Magnitude × Side interaction effect, *F*(1,23) = 0.85, *p* = 0.367. None of the other interactions were significant (all *p*s > 0.05). We thus replicated the findings of our Experiment 2, showing that number magnitude did not affect visual spatial attention. We further analyzed the interaction effects between magnitude and side using the BIC. The posterior probability favoring the null hypothesis was *p*_BIC_(H_0_ | D) = 0.76 for the two-way interaction between magnitude and side. This shows that there is more evidence for the null hypothesis than for the alternative hypothesis. Also, when we performed an analysis of Experiment 2 and Experiment 5 combined using the BIC. The posterior probability favoring the null hypothesis was *p*_BIC_(H_0_ | D) = 0.80 for the two-way interaction between magnitude and side. This confirms that there is more evidence for the null hypothesis than for the alternative hypothesis.

In order to test whether participants showed a SNARC effect on the parity decision we performed a 2 (Magnitude: high vs. low digit) × 2 (Side: left vs. right) × 4 (Delay: 250, 500, 750, or 1000 ms) repeated measures ANOVA with Group (congruent vs. incongruent response mapping) as between subjects factor (see Priftus et al., 2006). As in Experiment 2, we did not find a significant Magnitude × Side × Group interaction, *F*(1,22) = 0.02, *p* = 0.887. Also, the Magnitude × Side interaction remained non-significant, *F*(1,22) = 0.81, *p* = 0.378.

## REPLICATION EXPERIMENT 3

### RESULTS AND DISCUSSION

Trials with incorrect responses to either the target (1.46%) or magnitude judgment (2.59%) were excluded from the analysis. Trials with reaction times more than 2.5 standard deviations faster or slower than the subject’s mean reaction time were also excluded from analysis (5.17%). In **Figure 3C**, the reaction times to the target are plotted. In the left panel reaction times to targets presented at the left are plotted, in the right panel reaction times to targets presented at the right are plotted. The reaction times on the target task were submitted to a 2 (Magnitude: high vs. low digit) × 2 (Side: left vs. right) × 4 (Delay: 250, 500, 750, or 1000 ms) repeated measures ANOVA. Contrary to our expectations, we did not replicate the interaction effect for Magnitude × Side that we obtained in Experiment 3, *F*(1,23) = 0.00, *p* = 0.973. We found a main effect for Delay, *F*(3,69) = 27.07, *p* < 0.0001 and Side, *F*(1,23) = 6.71, *p* = 0.016. The main effect for Magnitude was not significant (*F* < 1). None of the other interactions were significant (all *p*s > 0.05).

We further analyzed the interaction effects between magnitude and side using the BIC. The posterior probability favoring the null hypothesis was *p*_BIC_(H_1_| D) = 0.82 for the two-way interaction between magnitude and side, which shows that there is more evidence for the null hypothesis than for the alternative hypothesis.

We also performed an overall analysis on the data from all three experiments; a 3 (Experiment: 1, 2, and 3) × 2 (Magnitude: high vs. low digit) × 2 (Side: left vs. right) × 4 (Delay: 250, 500, 750, or 1000 ms) repeated measures ANOVA. We did not find an Experiment × Magnitude × Side interaction effect, *F*(2,69) = 0.32, *p* = 0.729. This finding, combined with the BIC values of the three separate experiments, confirms that the effect of number magnitude on spatial attention was not present in any of the replication experiments.

Additionally, we performed an overall analysis on the interaction effects between magnitude and side for Experiments 3 and 6 combined using the BIC. The posterior probability favoring the null hypothesis was *p*_BIC_(H_1_| D) = 0.51 for the two-way interaction between magnitude and side, which shows that there is about as much evidence for the null hypothesis as for the alternative hypothesis. This confirms that the magnitude × side interaction effect is very fragile.

## GENERAL DISCUSSION

In the present study we tried to replicate the finding that merely perceiving a number affects spatial attention, an original effect found by Fischer et al. (2003). We investigated whether activation of the mental number line and subsequent direction of spatial attention in an image schema congruent manner was modulated by the relevance of magnitude information. In six (two sets of three) experiments, of which two were exact replications of the original study of Fischer et al. (2003) we manipulated the degree to which magnitude information was task-relevant. In five of the six experiments we obtained no effect of number magnitude on spatial attention. Even when participants processed the number in a parity judgment task, there was no effect on spatial attention. Only when participants actively processed number magnitude, by deciding whether the number was higher or lower than 5, we found in one of two experiments that attention was directed to the left by low numbers and to the right by high numbers. Thus, unlike Fischer et al. (2003) we did not find that (merely) perceiving a number-induced a shift of visual spatial attention. At best we found that when magnitude information is actively processed spatial representations associated with number meaning are activated, producing a corresponding shift in spatial attention.

The absence of image schema activation when participants merely perceived the numbers is in accordance with results from studies showing that instructional differences can impair a visual spatial attention effect (e.g., Galfano et al., 2006; Ristic et al., 2006). This indicates that activation of the mental number line is very sensitive to the relevance of magnitude. Even the original SNARC effect (in which the effect is found directly in manual responses to the numbers) is smaller when the number is processed only superficially (Fias, 2001; Wood et al., 2008). At first sight it might seem surprising that we also did not obtain a number-induced spatial attention effect when participants had to judge the parity of the number. Such results appear to contradict Casarotti et al. (2007) who showed that naming a number leads to a number-induced attention effect. However, one might argue that naming a number is more neutral than judging the parity of a number. When participants need to make a parity decision they attend to the parity feature of the number instead of magnitude, thus, making it more difficult to activate another feature such as magnitude. However, the results do not rule out that magnitude information is activated to some degree. For example, parity and magnitude might both be activated, but when a parity decision has to be made, magnitude information may be inhibited. This alternative mechanism is less likely, however, because it fails to explain why no effect was obtained in Experiment 1.

Not finding a number-induced attention effect when participants judge the parity of the number also appears to contradict findings with the original SNARC paradigm, in which researchers tend to obtain interaction effects of response side and number magnitude when participants make parity judgments. Note, however, that there is a fundamental difference between the underlying mechanisms of the original SNARC effect and the number-induced visual spatial attention effect. The original SNARC effect is found when participants make a response to the number itself. For example, in a parity judgment task, participants have to decide as quickly as possible whether the number is odd or even, thus the number itself is the target. Due to the requirement of binary, bimanual responses the response location becomes relevant, possibly activating a horizontal mental representation due to the horizontal response locations themselves (see Proctor and Cho, 2006, for a related argument). In studies examining the number-induced effect on spatial attention, however, there is no activation of spatial response codes because participants have to respond unimanually to an unrelated target. Thus, one could argue that spatial attention effects are more likely to reflect the underlying mental representation of numbers than the original SNARC effect, because the latter might additionally be influenced by response-related processing (e.g., Keus and Schwartz, 2005; Keus et al., 2005; Daar and Pratt, 2008).

The findings of Experiment 3, which we failed to replicate in Experiment 6, are somewhat consistent with studies showing order-related effects on spatial processing for other concepts that have an ordinal sequence, such as letters, days, and months (Gevers et al., 2003, 2004). Recently, it has been argued that ordinality may also drive spatial–numerical associations (Shaki and Gevers, 2011). As such, Dodd et al. (2008) showed that order-relevant processing leads to an attention effect. However, with respect to our current results, we cannot tease apart whether the number-induced attention effect in Experiment 3 is driven by magnitude (cardinality) or ordinality, because the mental number line in itself represents both ordinality and cardinality. Besides, our results indicate that the spatial image schema only occasionally affects attention when magnitude information is relevant since we could not replicate our own finding. The combined results of our six experiments suggests that the spatial image schema represents magnitude at best, but not other aspects of numbers, such as parity.

Not only context may explain the inconsistent findings in the literature concerning the number-induced attention effect, individual differences may also explain the inconsistencies. A recent line of research has shown that math proficiency modulates the strength of the SNARC effect irrespective of visual spatial working memory capacities (Hoffmann et al., 2014). These findings suggest that math proficient participants have automatic access to numerical representations, whereas non-proficient participants have difficulty in inhibiting irrelevant numerical information, such as magnitude information when performing a parity judgment. Other studies, however, have obtained no relation between math proficiency and the strength of the original SNARC effect (Cipora and Nuerk, 2013).

In five of the six experiments we found shorter reaction times for targets presented on the right side. These results correspond to work of Henderson (e.g., Henderson and Hollingworth, 1998) showing that participants prefer to look at the right side of the visual field. However, his preference is unrelated to the effect of number magnitude, and therefore cannot explain our lack of number-induced spatial effects on attention.

In conclusion, the current study provides very little evidence for an automatically activated mental number line. Such an effect would have been evidence for the conceptual metaphor theory, which states that metaphors provide grounding for abstract concepts by connecting them to more concrete domains. Fundamental for the conceptual metaphor theory is that image schemas are essential for the representation of abstract concepts. The results of our experiments demonstrate that activation of the mental number line is not automatic and might be sensitive to task-related importance of magnitude. Even when magnitude was task-relevant, we only found number-induced effects on spatial attention in one of our experiments, and could not replicate our own finding. This suggests that the effect is weak in relevant contexts and absent in all other contexts. Therefore, the mental number line does not seem to play an important role in number processing.

## Conflict of Interest Statement

The authors declare that the research was conducted in the absence of any commercial or financial relationships that could be construed as a potential conflict of interest.
