# Corrigendum: Number-induced shifts in spatial attention: a replication study

**DOI:** 10.3389/fpsyg.2014.01206

**Published:** 2014-10-27

**Authors:** Kiki Zanolie, Diane Pecher

**Affiliations:** ^1^Department of Psychology, Erasmus University RotterdamRotterdam, Netherlands; ^2^Institute of Psychology, Leiden UniversityLeiden, Netherlands

**Keywords:** corrigendum, Figure 1, placeholders, digit display, missing placeholders

This is a corrigendum for Number-Induced Shifts in Spatial Attention: A Replication Study.

Figure 1 of the manuscript does not completely represent the exact configuration of the experiments, such that the Display of the Digit should also hold two placeholders as in all the other Displays (Fixation, Variable delay, Target and Parity/Magnitude judgment). This is a subtle but critical difference.

**Figure 1:**
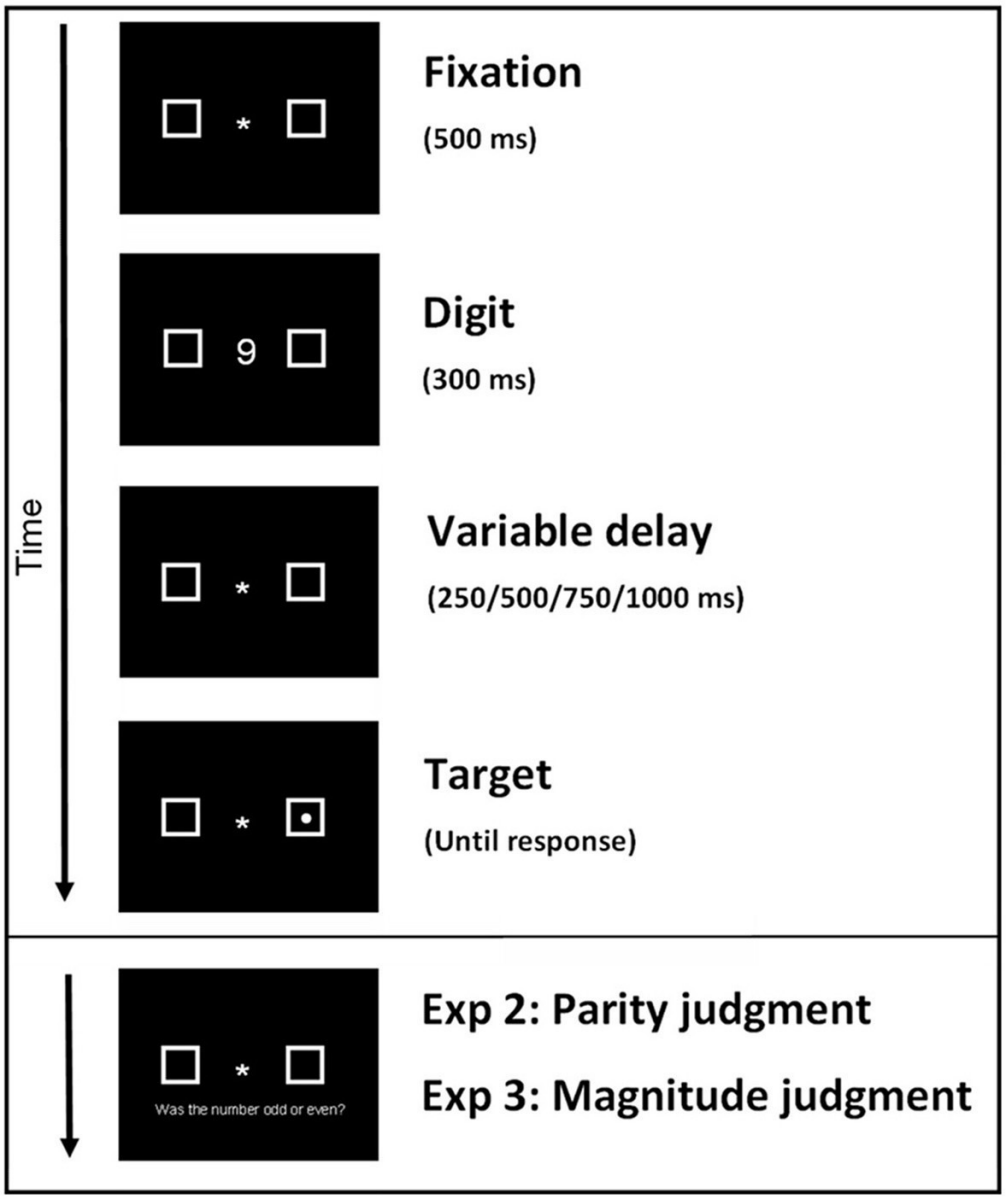
**The trial sequencestarted with a 500 ms fixation cross, followed by a 300 ms digit (1,2,8,9) display, and a variable delay of 250, 500, 750, or 1000 ms**. Then a target was presented randomly in one of the two place holders on 80% of all trials. Participants had to respond as fast as possible by pressing the space bar when they detected the target. In Experiment 2 and 3 a delay of 200 ms (as the variable delay) was presented after which participants decided whether the previously seen digit was odd or even (Experiment 2 and Replication Experiment 2) or whether the digit was higher or lower than 5 (Experiment 3 and Replication Experiment 3). Underneath the parity or magnitude question the response options with corresponding keys “z” and “x” where displayed.

## Conflict of interest statement

The authors declare that the research was conducted in the absence of any commercial or financial relationships that could be construed as a potential conflict of interest.

